# Candidate Genes and Pathways in Rice Co-Responding to Drought and Salt Identified by gcHap Network

**DOI:** 10.3390/ijms23074016

**Published:** 2022-04-05

**Authors:** Zhiqi Hao, Sai Ma, Lunping Liang, Ting Feng, Mengyuan Xiong, Shangshu Lian, Jingyan Zhu, Yanjun Chen, Lijun Meng, Min Li

**Affiliations:** 1 College of Agronomy, Anhui Agricultural University, Hefei 230036, China; 20721019@stu.ahau.edu.cn (Z.H.); 20721040@stu.ahau.edu.cn (S.M.); liang2505568381@stu.ahau.edu.cn (L.L.); 20720156@stu.ahau.edu.cn (T.F.); 17766105110@stu.ahau.edu.cn (M.X.); 21720167@stu.ahau.edu.cn (S.L.); zhujingyan@stu.ahau.edu.cn (J.Z.); chen21720131@stu.ahau.edu.cn (Y.C.); 2 Shenzhen Branch, Guangdong Laboratory of Lingnan Modern Agriculture, Genome Analysis Laboratory of the Ministry of Agriculture and Rural Affairs, Agricultural Genomics Institute at Shenzhen, Chinese Academy of Agricultural Sciences, Shenzhen 518120, China

**Keywords:** drought tolerance, salt tolerance, QTL/genes, gcHap-network, pathway, rice

## Abstract

Drought and salinity stresses are significant abiotic factors that limit rice yield. Exploring the co-response mechanism to drought and salt stress will be conducive to future rice breeding. A total of 1748 drought and salt co-responsive genes were screened, most of which are enriched in plant hormone signal transduction, protein processing in the endoplasmic reticulum, and the MAPK signaling pathways. We performed gene-coding sequence haplotype (gcHap) network analysis on nine important genes out of the total amount, which showed significant differences between the *Xian*/*indica* and *Geng*/*japonica* population. These genes were combined with related pathways, resulting in an interesting mechanistic draft called the ‘gcHap-network pathway’. Meanwhile, we collected a lot of drought and salt breeding varieties, especially the introgression lines (ILs) with HHZ as the parent, which contained the above-mentioned nine genes. This might imply that these ILs have the potential to improve the tolerance to drought and salt. In this paper, we focus on the relationship of drought and salt co-response gene gcHaps and their related pathways using a novel angle. The haplotype network will be helpful to explore the desired haplotypes that can be implemented in haplotype-based breeding programs.

## 1. Introduction

Rice (*Oryza sativa* L.) is the primary staple food crop for nearly half of the world’s population. Due to the drier and warmer climate trends, the amount of water used in agricultural irrigation is expected to increase [1], but limited available freshwater resources are likely to cause more severe drought pressure on crops. The Global Drought Information System (GDIS) reveals that drought is becoming progressively more severe and intense on a global scale [2]. Asia is the most important rice production area that is deeply influenced by drought stress. Agricultural zones in China are suffering from water shortages, with droughts occurring frequently. Since the 1990s, about 26 million hectares of arable land have been annually affected by drought, directly leading to a reduction in 70 million tons of food crops [3]. Similarly, in the Mekong River Delta (MRD), salt stress has also caused great economic losses. From 2015 to 2016, MRD experienced severe saltwater intrusion, causing a total of 215,445 hectares of rice to be severely affected [4]. Compared with the baseline period (2000), the sea level is expected to increase by 5 cm in 2050, and another 30,000 hectares of the agricultural area will be affected, which will cause serious economic losses [5]. Moreover, the pH of the soil has an influence on rice. For instance, the pH of the soil is controlled by the leaching of cations, such as Ca, Mg, K and Na, far exceeding their loss in weathered minerals, leaving H^+^ and Al^3+^ for the main cation exchange [6]. The most suitable soil condition for *Geng*/*japonica* is pH 4.0 [7], while for *Xian*/*indica* is at pH 5.0–5.5 [8]. The suitable pH can provide nutrients for plant growth, while an unsuitable pH can cause plant ion poisoning. Some salt-tolerant species can withstand higher soil pH environments up to 10 [9]. In recent years, some drought- and salt-tolerant rice varieties have been identified [10]. For example, Torres et al. (2013) screened the yield of 988 rice materials from the International Rice Research Institute (IRRI) under drought stress and finally identified more than 65 drought-tolerant rice materials, including Kataktara Da2, Shada Shaita and Dular [10,11,12]. There are also many well-known salt-tolerant varieties bred in south/east Asia, such as Kala Rata 1-24, Nona Bokra, Bhura Rata, SR 26B and Chin.13. [13] Many important QTLs, such as *Deeper Rooting 1* (*DRO1*), increased plant deep roots under drought stress and *SKC1* promoted Na^+^/K^+^ transport under salt stress [14,15]. Among them, many important genes have been successfully cloned and their functions have been identified. The functional genes, such as *D*, *OsHKT1;5*, were related to drought and salt stress, respectively [16]. Meanwhile, epigenetic (modifications) are instrumental in response to plant adversity stresses, involving histone modification, chromatin remodeling, non-coding RNAs and DNA methylation, and each play an important role in different epigenetic modifications. These modifications, which are single or in combination with each another, could affect gene expression and cause response to abiotic stress [17,18]. For instance, the expression of drought-related genes is closely associated with the alteration of histone dynamics, such as *RD29A*, *RD29B*, *RD22*, and *RAP2.4* [19]. In wild-type Arabidopsis, a putative small RNA target region was identified that negatively regulates Na^+^-selective transporter gene *AtHKT1* expression [19]. The mechanisms by which plants respond to drought and salt might be closely related [20]; however, analyses of drought and salt co-response mechanisms are rare, and most of these have focused on the function of genes responsive to a single stress. In this study, we collate drought and salt co-response genes and analyzed their related pathways using the genome-coding sequence haplotype (gcHap), thereby obtaining a mechanistic draft called the ‘gcHap-network pathway’. Here, we focus on a binding method to preliminary interpret the drought and salt co-response mechanism and apply it to rice breeding.

## 2. Phenology, Physiological, and Biochemical Indicators of Drought and Salt Tolerance 

**Figure 1:**
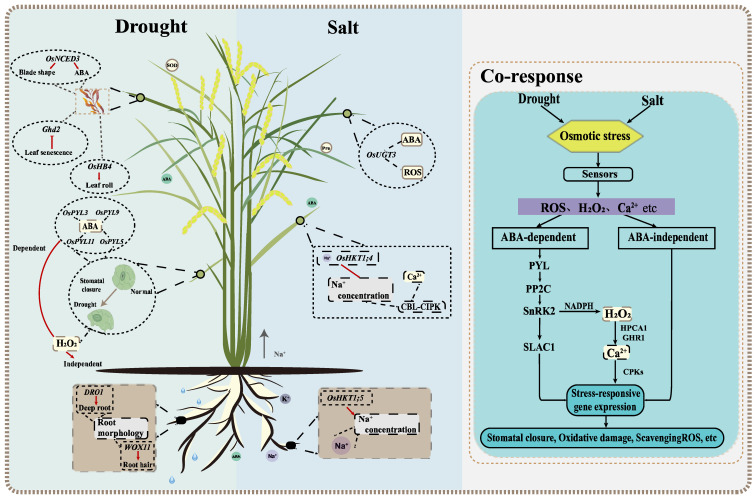
Diagram of reaction mechanisms involving drought and salt in rice. The left box of the larger picture is the regular mechanism of underground and aboveground parts under drought stress. Root composition can affect the water absorption and it is crucial to improve plant drought resistance. Leaf morphology and stomatal conductance of aboveground plants play a decisive role in water retention. The right box is the response mechanism under salt stress. The rejection of Na^+^ in roots can reduce the accumulation of Na^+^ in tissues. The response mechanisms of ion transport, reactive oxygen species and Ca^2+^ content in aboveground parts can effectively alleviate the effects of salt stress. The rightmost box shows the co-response mechanism under drought and salt stress. Some secondary messengers, such as Ca^2+^, and ROS can alleviate the damage of osmotic stress to plants and improve the drought and salt tolerance through ABA-dependent and ABA-independent pathways. The main components in the core ABA signaling transduction pathway include ABA receptor PYLs, SnRK2s, ABA co-receptor branch type A 2C protein phosphatase (PP2Cs) and SnRK2.

Generally, most studies of drought and salt stress focus on roots and shoots, which can be measured through phenotypic, physiological, and phenological parameters (Figure 1) [13,21]. Among them, ABA plays an important role in plant response to abiotic stresses, such as drought and salt [22]. The overexpression of *OsPYL5* can improve drought tolerance (DT) and salt tolerance (ST) through ABA-mediated processes [23]. Studies have shown that ABA exists in the roots and shoots of plants. Huang et al. (2010) overexpressed the rice NCED gene *OsNCED3*, promoted ABA synthesis in Arabidopsis, changed leaf morphology and reduced water loss [16]. Huang et al. (2021) found that salt-stress-induced ABA-response gene expression and ABA accumulation in rice roots, thereby increasing plant sensitivity to salt [24]. Since roots are responsible for the absorption and transportation of water and nutrients, root morphological and physiological characteristics play a decisive role in rice yield under drought conditions [25]. In addition to ABA, rice can also change root morphology and increase water absorption in an ABA-independent way under drought stress. One of the best examples of drought avoidance QTL is *DRO1*, which increases the deep roots by participating in the elongation of root tip cells [14]. Another noteworthy example is that the WOX11-ERF3 interaction induces epidermal cells to differentiate into root hairs, thereby promoting water and mineral absorption by increasing root biomass [26]. Some genes related to rice leaf area index, chlorophyll content and leaf relative water content have also been reported. These genes are helpful to study rice leaf traits under drought stress. *GHd2* regulates leaf senescence under drought stress by interacting with different proteins; *OsHB4* overexpression can alter leaf morphology and reduce water loss; Gao et al. (2020) found that *OsGUX1* affects relative chlorophyll content in rice leaves [27,28,29]. At the same time, drought-tolerant rice varieties, such as Indian local varieties Kalajeera, Machakanta and Haladichudi, can also be distinguished by leaf traits [30,31,32]. 

Salt stress causes changes in plant osmotic and ionic pressure, which inhibits the plant growth. Recent reports suggest that “ionic imbalance” is closer to an ionic unstable state. The excessive accumulation of Na^+^ and Cl^-^ affects the influx and metabolism of other ions, which in turn affects plant growth and development [33]. It illustrates the important sensing mechanism that initiates a series of transduction pathways in rice that hinders plant height, development, metabolism, ion accumulation, and other characteristics (Figure 1) [34]. Rice perceives salt stress initially as Na entering the root system via nonselective cation channels (NSCCs), then being sensed by the leaves through the long-distance transport [34]. In rice, Na^+^ content, the alteration of intracellular Ca^2+^ levels, and the accumulation of reactive oxygen species (ROS) are the initial signal responses to salt stress [35,36]. Na^+^, K^+^ content, and Na^+^/K^+^ ratio are important indexes for measuring rice ST [37]. High-affinity K^+^ transporters (HKT) can effectively stabilize Na^+^ at the cell level [38]. *OsHKT1;5* excretes Na^+^ from the roots’ xylem parenchyma cells (XPC) [39]. Furthermore, *OsHKT1;4* can inhibit the Na^+^ content in the leaves at the reproductive growth stage [40]. Meanwhile, the CBL–CIPK calcium signal network plays a key role in sensing salt-induced Ca^2+^ signals and regulating Na^+^/K^+^ ion homeostasis [41]. In response to increased ROS, rice plants decompose ROS through a series of biochemical reactions and antioxidant enzymes [42]. Therefore, the activities of these enzymes can be used as an effective criterion for ion toxicity under salt stress. *OsUGT3* participates in dynamic changes in ABA, effectively reducing ROS toxicity by increasing antioxidant enzyme activity [43].

There are many similarities between the evaluation indicators of DT and ST (Figure 1). In general, after the recognition of the receptor stress signal from the cell membrane, plants carry out signal transduction by secondary messengers, such as ROS, Ca^2+^, and initiate downstream stress-response gene expression through ABA-dependent and ABA-independent pathways [44]. The accumulation of ions in rice under salt conditions leads to water deficiency in vivo and ultimately similar stress responses to drought. These reactions include the adaptive response, the accumulation of various osmotic agents and antioxidants, intracellular Ca^2+^ spikes, and a large amount of ROS accumulation [41]. The osmotic pressure can be relieved by the accumulation of proline and soluble sugars. Increased ABA content induces stomatal closure and reduces water loss in guard cells, which has a significant impact on osmotic potential reduction [45]. The ABA signaling pathway, acting as a pivotal process in biotic and abiotic stress responses, plays an important role in drought and salt responses [38,46]. The main components in the core ABA signaling transduction pathway include ABA receptors PYR/PYL/RCARs, SnRK2s and ABA co-receptor branch type A 2C protein phosphatase (PP2Cs) [41,47]. In addition, H_2_O_2_ plays an important role in stomatal closure through ABA-dependent and ABA-independent pathways [38]. In the ABA signaling pathway, ROS is activated by SnRK2 in NADPH oxidase and promotes the production of H_2_O_2_ [48]. Moreover, H_2_O_2_ is perceived by leucine-rich repeat receptor kinases HPCA1 and GHR1, which activate the Ca^2+^ channel and affect stomatal closure through Ca^2+^-dependent protein kinases (CPKs) and SAC1 [49]. Under drought and salt conditions, stress-response genes increase plant resistance through the activation of related proteins and the accumulation of protective metabolites. By inhibiting the expression of *DST* and *ABIL2*, *HDA704* positively regulates rice DT and ST [22,50].

Accordingly, drought and salt stress can affect the growth morphology, physiological and biochemical indicators, molecular characteristics and yield of rice. Most studies elaborate on the drought and salt regulation mechanisms, but in many places, the lands are currently affected by multiple types of coercion. It is important to understand the co-response mechanism under drought and salt stress, which can be used as a basis for the selection of multiple tolerant varieties and as an effective way to promote rice DT and ST breeding.

## 3. Genetics of Drought Tolerance and Salt Tolerance in Rice

According to the common response of plants to drought and salt stress, the mapping of QTLs and/or genes related to both DT and ST in rice is of great significance for the design of rice stress-resistance breeding programs. At present, most of the drought-resistant QTLs in rice are related to important traits, such as yield, shoot and root growth, osmotic adaptation, hormone response and photosynthesis [51]. The QTLs for salt resistance are determined according to ST score (STS), aboveground potassium concentration, sodium concentration, chlorophyll content and dry weight [52]. In this paper, many DT and ST QTLs/genes were sorted through the study of rice under different genetic backgrounds and environments [53].

### 3.1. Collection of Cloned QTLs/Genes

A total of 392 drought-related QTLs were obtained that are involved in plant height, leaf roll rate, yield, root dry weight, root penetration index, biomass and other important traits (Supplemental Table S1). We collected 435 QTLs of salt sensitive varieties and ST varieties from previous studies (Supplemental Table S2) [53]. For salt-related QTLs, the largest number of QTLs was associated with ion concentration traits under salt stress, as this is beneficial to maintaining stable metabolic processes in plants. By comparing the QTL mapping results of DT and ST, 25 QTLs were found to be co-response to drought and salt; there were numerous overlapping sites on chromosome 3 with 4 QTLs (Supplemental Table S3). The most common QTLs for drought and salt were related to plant height and leaf damage level. It was speculated that these loci may be caused by multiple effects or genetic overlap between different traits.

The identification of QTLs associated with drought and salt traits in rice contributed to the breeding of stress-resistant varieties [54]. Cui et al. [54,55] used the selective breeding population as a recurrent parent to compare the QTL mapping results under drought and salt conditions, and found that they overlapped at 78cM on chromosome 1. Using the BC_2_F_8_ introgression line (IL) populations of IR64/Binam and Teqing/Binam, five and three overlapping QTLs related to Na^+^, K^+^ content, thousand grain weight (TGW) and plant height (PH) were detected for drought and salt stress, respectively [56]. Using backcross IL populations, *RM231* and *RM335* were used to locate two genetic overlap sites of DT and ST [57]. This confirmed the existence of the same QTLs existed in the DT and ST of rice, as well as the existence of partial genetic overlap in DT- and ST-regulated mechanisms. The molecular screening of important rice DT genotypes by QTL combined with simple sequence repeat (SSR) and single nucleotide polymorphism (SNP) markers can quickly and accurately analyze rice lines. Pang et al. [57,58] located the QTLs of yield and related traits in upland, salt and paddy fields by selecting breeding populations. Using high-density SNP marker information from the sequencing of 3000 rice genomes, 15 candidate genes were found in the QTL region [57,58].

In addition, 798 genes were screened, including 361 drought-related, 437 salt-related, and 161 drought and salt co-response genes (Supplemental Tables S4–S6). Drought- and salt-related genes were found to be the most numerous among all known co-response genes on chromosomes 1, 2 and 3, followed by chromosomes 4, 5, 6 and 7. Notably, drought- and salt-related QTLs and genes are concentrated on chromosomes 1 and 2. It is speculated that the alleles of these loci have a positive effect on rice yield under drought and salt stress. Drought and salt co-response genes are also mainly distributed on chromosomes 1, 2 and 3, which can be divided into regulatory genes and functional genes. The genes regulated by the transcription factors, as regulatory genes, which can regulate gene expression, are effective candidate genes for enhancing crop stress tolerance [59]. Many DT and ST co-response genes belong to transcription factor families, including MYB, NAC, bZIP, and zinc finger proteins. MYB is one of the largest transcription factor families and plays an important role in hormone signal transduction under stress and in biological and abiotic responses [60]. In addition, studies have shown that the transcription factor Cdt-NF-YC1, as a candidate gene, can enhance plant DT and ST through modulating gene expression in both the ABA-dependent and ABA-independent pathways [61]. *OsHBP1b* belongs to the bZIP family, which enhances rice stress tolerance by improving antioxidant enzyme activity and reducing ROS damage [62]. Protein kinase signal transduction plays an important role in plant stress. *OsCDPK7* is a Ca^2+^-dependent protein kinase that enhances DT and ST in rice when overexpressed [63].

### 3.2. Data Acquisition and Analysis by Public Databases

A total of 11,688 differentially expressed genes (DEGs) were induced by drought and salt stress, including nine materials identified in Plantsexpress (http://plantomics.mind.meiji.ac.jp/OryzaExpress/, accessed on 1 February 2022) using the 4 × 44 K Microarray (Supplemental Table S7). Under drought stress, there were 6742 DEGs, including 2930 up-regulated genes. A total of 7328 DEGs were related to salt stress, including 3729 up-regulated genes. Under drought or salt stress, the heatmap of all DEGs demonstrated that the expression levels of some genes decreased compared with the control in the leaf or root (Supplementary Figure S1A,B). From the sample above we found 1772 co-response genes (694 up-regulated and 1078 down-regulated). Notably, some genes were up- and down-regulated simultaneously under drought or salt stress, or perhaps they were up-regulated under drought and down-regulated under salt. Although different samples might cause this phenomenon, the results of up- and down-regulated genes were generally in line with expectations under drought and salt stress.

## 4. Gene–CDS–Haplotype and Functional Analysis of the DT and ST Genes in Rice

### 4.1. Gene Ontology and Pathway Analysis

To reveal the DT and ST co-response functions of each co-response module, we further analyzed the cloned genes and DEGs using PlantGSEA and OmicShare tools [64], which are based on the Gene Set Enrichment Analysis (GSEA) and Kyoto Encyclopedia of Genes and Genomes (KEGG) databases [65]. Combining the results of previous studies and public databases, we then characterized 6019 (drought related) and 6493 (salt related) genes to identify the three processes, namely biological processes (BP), cellular components (CC) and molecular function (MF), that were assigned. First, for drought-related genes, they were significantly enriched in biological regulation (GO:0065007, *p* = 6.60 × 10^−6^), organelle (GO:0043226, *p* = 4.20 × 10^−8^) and transporter activity (GO:0005215, *p*= 0.169238206) (Supplemental Figure S2A). The results of salt-related gene enrichment are similar to those of drought (Supplemental Figure S2B). Second, we focused on the co-response genes under drought and salt stress, which were significantly enriched in GO:0008152, GO:0044464 and binding (GO:0005488), respectively (Supplemental Table S8). In addition, 723 genes were up-regulated, most of which were mostly related to metabolic processes, chloroplasts, and transporter activity. Small molecule metabolic process (GO:0044281, *p* = 1.3 × 10^−5^), chloroplast (GO:0009507, *p* = 9.6 × 10^−9^) and plastid (GO:0009536, *p* = 9.6 × 10^−9^) were the most significantly enriched GO terms (Supplemental Figure S2C). For 1025 down-regulated co-response genes, most of them were related to drought and salt stress, such as water, abscisic acid and mRNA processing, and were significantly enriched in response to water deprivation (GO:009414, *p* = 9 × 10^−5^), mRNA metabolic process (GO:0016071, *p* = 9 × 10^−5^) and intracellular process (GO:0005622, *p* = 5 × 10^−4^) (Supplemental Figure S2D). Third, we chose the above drought and salt co-response genes to perform the KEGG analysis. In total, 20 pathways were significantly enriched, which were mainly enriched at osa01100, osa01110 (metabolic pathways, biosynthesis of secondary metabolites; 142/65 genes, *p* = 1.83 × 10^−36^/1.43 × 10^−14^), osa04075 (plant hormone signal transduction; 26 genes, *p* = 4.32 × 10^−12^), osa04141 (protein processing in endoplasmic reticulum; 25 genes, *p* = 5.47 × 10^−9^) and osa03040 (spliceosome; 23 genes, *p* = 6.21 × 10^−8^) (Table S9, Supplemental Figure S3). Using the public databases, the genes enriched at osa04075 were then processed under different phytohormone treatments, which revealed that these genes were significantly induced by ABA in the shoots and roots (Supplemental Figure S4). The signals carried by the pathway networks were different from those in the osa04075-enriched gene points of signal transduction, thereby acting as molecular switches for drought and salt stress (Supplemental Figure S5A). In particular, the *OsHSPs* gene family played essential roles in primary metabolism processes and signal transduction, which were enriched at osa04141 and osa03040, and were related to abiotic stress (Supplemental Figure S5B).

### 4.2. GcHap Diversity of Genes Positively Regulated by DT and ST

**Figure 2:**
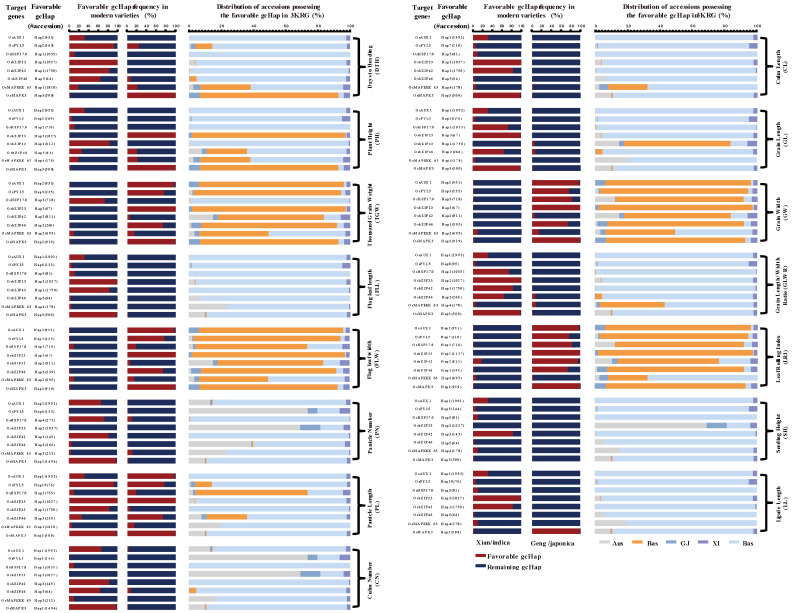
Frequencies of the ‘‘favorable’’ gcHaps of nine drought and salt co-response genes affecting fifteen yield traits (DTH, PH, TGW, FLL, FLW, PN, PL, CN, CL, GW, GLWR, LRI, SH and LL) in *Xian*/*indica* (XI) and *Geng*/*japonica* (GJ) modern varieties and different rice populations.

After the DT and ST co-response genes were subjected to the GO and KEGG analysis, nine of them were up- /down-regulated and cloned genes, which were chosen to perform the frequency of ‘favorable’ gcHaps affecting yield-related traits and different populations. In the RFGB database (https://www.rmbreeding.cn/Index/, accessed on 10 February 2022), the ‘favorable’ gcHaps of the DT and ST genes are defined as those associated with the highest traits value in phenotypic dataset [66]. Additionally, then 15 important yield traits are chosen, which shows the frequencies of favorable gcHaps at nine DT and ST co-response genes in 3,000 Rice Genomes Project (3KRGP) haplotypes (*n* > 50) (Figure 2). From this figure, it could be found that, firstly, the frequency of MV-*Xian* was a major character in 3010 rice genomes (3KRG) at most genes for 15 traits, such as panicle number (PN), panicle length (PL), culm length (CL) and grain length/width ratio (GLWR), whereas the target grain length (GL) and grain width (GW) values varied among specific *Xian*-*Geng* breeding efforts. Among them, the Hap3 of *OsMAPKKK63* and *OsbZIP42* was mostly predominant in *Aus* for PN and culm number (CN), whereas the favorable gcHaps of these genes were present at high frequencies in MV-*Geng*. Second, the most favorable gcHap at *OsbZIP23* is from MV-*Xian*. Due to the different favorable gcHaps for 15 traits, *OsPYL5* constituted a very low proportion in MV-*Xian* and MV-*Geng*. Compared with *OsPYL5*, *OsAUX1*/*OsHSP17.0* were present at high frequency in MV-*Xian*/*Geng*, which has only 3/5 haplotypes in 3KRG. These results suggest that most MVs of tolerance breeding currently were chosen in agricultural fields (Figure 2).

From these results, nine DT and ST co-response genes had favorable gcHaps distributed in the *Xian*, *Geng*, *Aus*, *Basmati (Bas)*, and *Admixtures (Admix)* populations in the 15 yield-related traits. The particular point is that the two haplotypes of *OsHBP1b* were filtered because their MAF (Minor Allele Frequency) was below 0.001.

### 4.3. gcHap-Network Pathway Analysis

**Figure 3:**
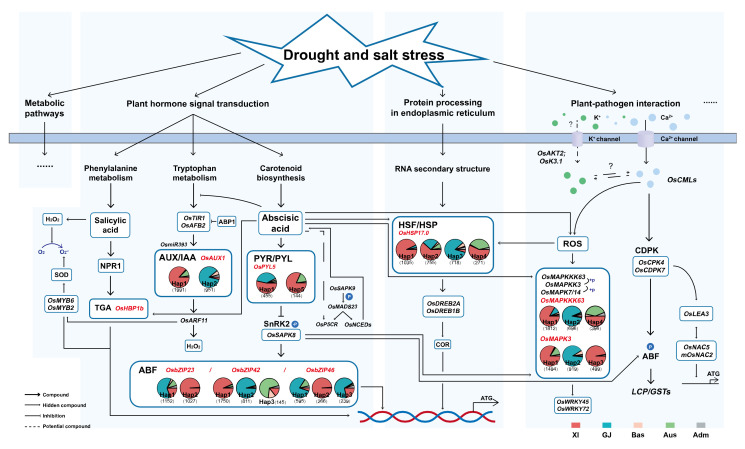
Dissection of gcHap-network pathway of important drought and salt co-response genes. These gene-related pathways and elite haplotypes for drought and salt co-response genes are indicated in the blue box. Firstly, *OsAUX1*, *OsPYL5*, and *OsbZIPs* (3 genes) were enriched in osa04075 that mainly included the auxin (AUX), ABA, and salicylic acid (SA) related pathways. The two rice auxin receptor genes *OsTIR1* and *OsAFB2* were targeted by *OsmiR393*, and the MYB transcription factor *MYB77* interacted with both *IAA19* and ARFs. H_2_O_2_ has been found to be involved in the morphogenesis of ARF. ABA occupies an important position in plant growth and plant development, and the components of the ABA signaling pathway mainly include *OsPYLs*, *OsPP2C*, *OsSAPKs*, and some transcription factors (TFs). Secondly, RNA secondary structure was influenced via the osa03040 or another pathway, which changed the activity and expression of HSF associated genes. Moreover, *OsHSPs* are associated with primary metabolism and signal transduction. Thirdly, the interaction of the Ca^2+^ and ROS signaling pathways has an important role in the regulatory network of plant immunity. Component changes in the MAPK cascade pathway were influenced by the activation of the ROS pathway. The PYL-PP2C-SnRK2 core ABA-signaling module activates a MAPK cascade, which may regulate many ABA effector proteins through phosphorylation. The MAPK cascade could also be activated by the PYL-PP2C-SnRK2 core ABA signaling module, which regulates ABA-related proteins through phosphorylation.

To excavate the important mechanism for drought and salt stress co-response and its application in breeding by design (BBD), we performed a gcHap network analysis on eight important genes illustrating the population structure and evolutionary relationships of major haplotypes (Supplemental Figure S6) and combined them with the related pathways, which obtained an interesting mechanistic draft called ‘gcHap-network pathway’ (Figure 3). Due to the incomplete haplotype sequences affecting the normal operation of the R package ‘pegas’ [67], the haplotypes of some genes are not shown in the figure.

First, the co-response genes enriched in osa04075 mainly included the auxin (AUX), ABA, and salicylic acid (SA) related pathways. AUX and IAA play critical role in drought stress response [68]. The auxin transport gene *OsAUX1* has two major gcHaps. Hap1 has the highest frequency in LAN-*Xian* and is present almost exclusively in *Xian*. Hap2 differed from Hap1 by three non-synonymous mutations, which were mainly distributed in *Geng* and was the favorable gcHap in days to heading (DTH), PH, and TGW. Both *OsAUX1* and sensing gene *OsTIR1* were identified in significant marker-trait association (MTA) regions [69]. Meanwhile, an auxin receptor, transport inhibitor resistant1 (*OsTIR1*) and a tiller inhibitor *OsAFB2* were down-regulated with the overexpression of a microRNA, *OsmiR393*, which was the target of two auxin receptor genes [70,71]. Auxin regulates the transcription of downstream genes in combination with AUX/IAA transcriptional suppressors to F-box TIR1/AFB proteins in the SCFTIR1/AFB complex [72].

ABA occupies an important position in plant growth and plant development. The family of *OsPYLs* is believed to be the largest plant hormone receptor family. In Arabidopsis, ABA could combine with PYLs and form PP2Cs [73]. *OsPYL5* possessed seven different favorable gcHaps, the frequency of which was observed to be similar between MV-*Xian* and MV-*Geng*. Hap1 was almost present in *Xian* and *Geng*, but Hap5 was predominant in *Xian* and *Aus*. In response to the presence of ABA, the activity of SnRK was affected by the PP2Cs, and the SnRK kinases phosphorylate their target protein in response to abiotic stress and affect the expression of downstream genes [74]. *OsPYL5* was also found in the pathway of osa04010 (MAPK signaling pathway). Some mitogen-activated protein kinase (MAPK) cascades play an important role in phytohormone accumulation, signal response, and plant growth and development [75,76,77]. *OsMAPK* cascades participate in some phytohormone responses, such as SA, jasmonic acid (JA) and ABA [75]. In rice, there are several osmotic stress/ABA-activated protein kinases, which are known as *SAPK1–10* [78]. In some studies, *SAPK6* and *OsbZIP46* co-response enhanced ABA and drought stress, and in other studies, *SAPK9* was shown to activate *OsbZIP46* under abiotic stress [78,79,80]. *OsbZIP46* had four high-frequency gcHaps and three minor derived ones: Hap1 and Hap3 (with Hap6, 7 derived) had a high frequency in *Geng*, *Aus*, and was the predominant gcHap in MV-*Geng*. By contrast, Hap2 (with Hap5 derived) was predominant in *Xian* and replaced Hap1 as the predominant gcHap in MV-*Xian*. *OsbZIP42* was a typical *Xian*-*Geng* divergent gene: Hap1 was predominant in *Xian* whereas Hap2 was predominant in *Geng*. The strong *Xian*-*Geng* differentiation was clearly reflected by the association of Hap1 with 15 yield-related traits. Similar to *OsbZIP42*, *OsbZIP23* had three major haplotypes; the differentiation of *Xian*-*Geng* was clearly reflected by differences between the frequencies of Hap1/Hap3 and Hap2. 

SA plays an important role in the regulation of plant innate immunity; H_2_O_2_ was found to connect with SA, which could further influence the downstream regulatory network [81]. In mung beans, SA and H_2_O_2_ were verified to be associated with auxin response factor (ARF) and transcription factor genes in the ROS oxidative response [82,83].

Second, osa04141 and osa03040 were the prevailing upregulated pathways of rice in ER stress response, which were linked with drought and salt tolerance [84]. RNA secondary structure was influenced via the osa03040 or another pathway, which changed the activity and expression of HSF associated genes [85]. Moreover, *OsHSPs* are associated with primary metabolism and signal transduction [86]. It was reported that *OsHSPs* played the role of ABA reporter and induced accumulation under drought and salt stress. Additionally, then, *OsDREBs* and *OsDREB2A* were regulated by *HSP*/*HSF* or ROS, which were also associated with drought and salt stress. For a good example, *OsHSP17.0* was up-regulated genes enriched at osa04141, and it possessed five different favorable gcHaps, the frequency of which was observed to be similar between MV-*Xian* and MV-*Geng*. Hap1 (with Hap4 derived), Hap2 (with Hap5, 6 derived) were mainly present in *Xian*, but Hap3 was predominant in *Geng*. Hap3 differed from Hap1/Hap2 by a single non-synonymous mutation.

Interestingly, some important genes, *OsMAPK3* and *OsMAPKKK63*, which have been shown to respond to drought and salt stress [75,87,88], were discovered in osa04626. This pathway might be associated with drought and salt stress, which have rarely been reported. First, these enriched genes were associated with Ca^2+^ signaling and are well known in the salinity response [34] as well as drought stress [89]. In Arabidopsis and rice, Ca^2+^ signals are decoded and transmitted by calcium-dependent protein kinases (CDPKs) and calmodulins (CaMs) [90]. In addition to the response to Ca^2+^ signals, *OsCDPKs* are also involved in ABA signaling. The interaction of the Ca^2+^ and ROS signaling pathways plays an important role in the plant immunity regulatory network [91]. Component changes in the MAPK cascade pathway were influenced by the activation of the ROS pathway [92]. The MAPK cascade could also be activated by the PYL-PP2C-SnRK2 core ABA signaling module, which regulates ABA-related proteins through phosphorylation [46]. In the Ca^2+^ channel pathway, *MPK3/6* was associated with CaMs, which included the drought- and salt-response gene *OsMAPK3*. *OsMAPK3* had three major gcHaps and two minor derived gcHaps, which had typical *Xian*-*Geng* divergent: Hap1 and Hap3 were predominant in *Xian* whereas Hap2 was mostly present in *Geng*. Hap2 differed from Hap1 by a single non-synonymous mutation. *OsMAPKKK63* had three major gcHaps: Hap1 was predominant in *Xian*, whereas Hap2 had a high frequency in *Geng*. Hap3 differed from Hap1 and Hap2, as preference existed in *Xian* and *Aus*. It was reported that overexpressing the downstream *WRKYs* (*OsWRKY45* and *OsWRKY72*) could enhance DT and ST in rice [93]. In addition, the K^+^ channel gene *LOC4338867* (*OsAKT2*) could combine with *OsCMLs* in the regulation of plant–pathogen interactions, but its position and relationship with the calcium channel are unknown [94]. 

Among the results we obtained, *LOC_Os02g35180* was up-regulated in signal transduction under drought and salt stress, an aspect not mentioned in previous studies. The link between reported regulated and unreported new genes is unknown, and the function and position of new genes need to be further elucidated. Although *OsHSP23.7* was up-regulated gene enriched in osa04141, osa03040 and response to drought and salt stress, its record was discontinued in National Center for Biotechnology Information (NCBI) database. Its KEGG pathway analysis did not proceed. Furthermore, the mechanism by which the regulatory mechanism of AUX/IAA regulates for abiotic stress (such as drought and salt) is unknown [27,95]. AUX can promote root elongation under salt stress, while *OsAUX1* is a down-regulated gene in the transcriptome data, which is consistent with our experimental results. MODD promotes the degradation of *OsbZIP46* by down-regulating its histone acetylation level to reduc drought resistance in rice. From previous studies and our results, *OsbZIP42*, *OsbZIP46*, and *OsbZIP23* exhibit significant differences in expression, which may be influenced by some factors, such as MODD, which in turn leads to down-regulated expression. Drought and salt tolerance related with local changes in gene expression in specific cell position, and in many cases gene expression can up- and down-regulated in different cell types in the same organ. Moreover, that most rapid and important response to stress occurred on epigenetic and protein activity levels, what serve as upstream regulator of stress tolerance. Chromatin status (such as methylation, acetylation, etc.) changes differently in different cell types and serves as “coordinator” of the certain pathways. For instance, *bZIPs* are associated with DNA methylation and chromatin remodeling under drought and salt stress, respectively [96]. Recently, the MAPK cascades have been reported to regulate plant immunity by participating in histone acetylation, and *MPK3* acts as a key regulator in histone modification-mediated chromatin regulation [97]. Meanwhile, some miRNAs are involved in the plant regulatory mechanisms reprogramming gene expression, such as *OsmiR393* [98]. In rice, although a number of DT and ST genes have been cloned and the effects of epistatic modifications on gene expression have been revealed, they may have different effects in different cell types. Gene expression at different sites is influenced by epistatic modifications, chromatin interactions, and the initial transcriptional event may serve as a target site for subsequent binding by other modifying proteins, which leads to nuclear structural reorganization and transcriptional activation of genes at other loci [99]. Finally, to explore the regulation mechanisms of DT and ST co-response genes and tap the impact of epigenetic modifications on them, there are still many questions and challenges to be addressed.

## 5. Breeding Approaches of Drought and Salt Tolerance

### 5.1. Improving DT and ST by Conventional Breeding Approaches

Conventional breeding methods, such as hybridization, backcrossing, and induced mutations, have been applied to the development of DT and ST rice. For varieties that do not have DT and ST among the existing popular varieties, molecular marker-assisted selection (MAS) can be carried out by selecting salt and drought genes to improve the stress resistance of existing varieties. The fine mapping of QTLs controlling abiotic stress tolerance can be used as the first-order trait of MAS. Therefore, MAS backcrossing (MABC) technology has been popularized as a method for obtaining beneficial QTLs and incorporating them into genotypes by using foreground markers of target loci and background markers of the rest of the genome [100]. The marker-assisted breeding program at IRRI has identified 12 major drought yield QTLs in the background of high yielding varieties IR64, et al. [101]. Additionally, scientists deployed the QTLs (*qDTY1.1*, *qDTY2.1*, *qDTY3.1*, *qDTY6.1* and *qDTY12.1*) through molecular breeding to develop and release a DT version of IR64, named DRR Dhan 42, DT MR219 and DT Sabitri [102]. There are 52 ST QTL studies on rice, and more than half of them are at the seedling stage [103]. In particular, considerable progress has been made in mapping the main QTL *Saltol*, which also provides an opportunity for the introduction of the *Saltol* QTL into 37 popular varieties in Bangladesh, India, Vietnam, and the Philippines. At least four popular varieties obtained ST QTLs through MABC [104]. In Vietnam, scientists developed IR64 [105], Bacthom 7 [106,107,108], Q5DB, and AS996 [109,110] using MAS, and the ST of varieties is controlled by the main *Saltol* QTL. Through cooperation between IRRI and BRRI, *Saltol* has been introduced into BR11, BRRI dhan28, BRRI dhan29, and IR64 and has been tested in the salt-affected coastal areas of the Philippines, Bangladesh, and other countries.

### 5.2. Improving DT and ST by Selective Introgression 

Traditional breeding consumes a lot of manpower and material resources, and it generally takes 6–8 years to cultivate an excellent variety, making it difficult to meet the needs of rice breeding. Because complex quantitative traits (such as yield and abiotic stress) are generally controlled by multiple genes, and are also affected by genetic background and environment, traditional breeding results are difficult to be directly applied in rice breeding [101]. Li et al. [102] proposed the concept and strategy of breeding by selective introgression (BBSI) for genetic analysis of complex traits and improvement of new varieties. We searched for information on 888 rice accessions from the literature, of which 573 were ST varieties, mainly from Asia. China and India were the main sources, with 165 and 184 varieties, respectively. The other 315 were DT varieties, 237 of which were from Asia, of which 207 were from China. Among these, 19 varieties originated from the IRRI (International Rice Research Institute) and were used as the parents of introgression lines (ILs). In our experiment, the DK population of 26 ILs was selected by the recurrent parent (IR64) and donor (Binam, BR24, OM1723, and HAN) through backcrossing and drought resistance. As an example of Huanghuazhan (HHZ) introgression, by way of DNA sequence alignment, we found that the nine co-response genes were present in HHZ and its donors, which might imply that the materials had the ability and potential to improve abiotic stress tolerance. Further, we sort out drought and salt tolerant varieties, many of which are in Green Super Rice (GSR), especially in the famous HHZ ILs. HHZ was improved by GSR, and in addition to HHZ, GSR also improved 66 rice varieties with various combinations of beneficial agronomic characteristics in China [111,112].

## 6. Conclusions, Challenges, and Prospects

We acquired 1748 drought and salt co-responsive genes based on QTL and transcriptome analysis; 1748 were significantly enriched in GO:0065007, GO:0043226 and GO:0005215, whilst nine important genes (*OsPYL5*, *OsMAPK3*, *OsbZIPs*, etc.) were selected and constructed the networks based on the RFGB database, with a total of 70 gcHaps (33 of which were incomplete haplotype sequences and could not be drawn), which had significant differences in *Xian*, *Geng*. We combined these gcHaps with the related pathways, making an interesting mechanistic draft called ‘gcHap-network pathway’. Furthermore, we sort up drought and salt tolerant varieties, especially in the famous HHZ ILs and their donors, including most important genes/alleles above. These results provide effective information for breeding for multiple resistance.

There have been a series of reports on drought and salt co-response gene functions that have made significant contributions. However, few studies have been conducted on haplotype population differences and the networks associated with them. Among the genes mentioned above, we focus on the drought and salt co-response genes based on the gcHap network, probing the relationship between these gcHaps and related pathways using a novel angle. To elucidate the mechanisms of abiotic stress, more analytical approaches, such as genomics, proteomics, metabolomics, and phenomics, should be combined in the future, and it is critical to keep innovating and developing more tolerant varieties. As previously stated, there is potential for the future development of stress-tolerant and high-yielding rice varieties.

## Data Availability

All of the data generated or analyzed during this study are included in this published article.

## Supplementary Materials

The following are available online at https://www.mdpi.com/article/10.3390/ijms23074016/s1.
