# Supplementary material for: Candidate Genes and Pathways in Rice Co-Responding to Drought and Salt Identified by gcHap Network
Source: Int J Mol Sci. 2022 Apr 5;23(7):4016. doi: 10.3390/ijms23074016 (PMC8999833; doi:10.3390/ijms23074016)
Supplement: Supplementary file 1 [file ijms-23-04016-s001.zip › Supplemental figure S1-6.pdf]

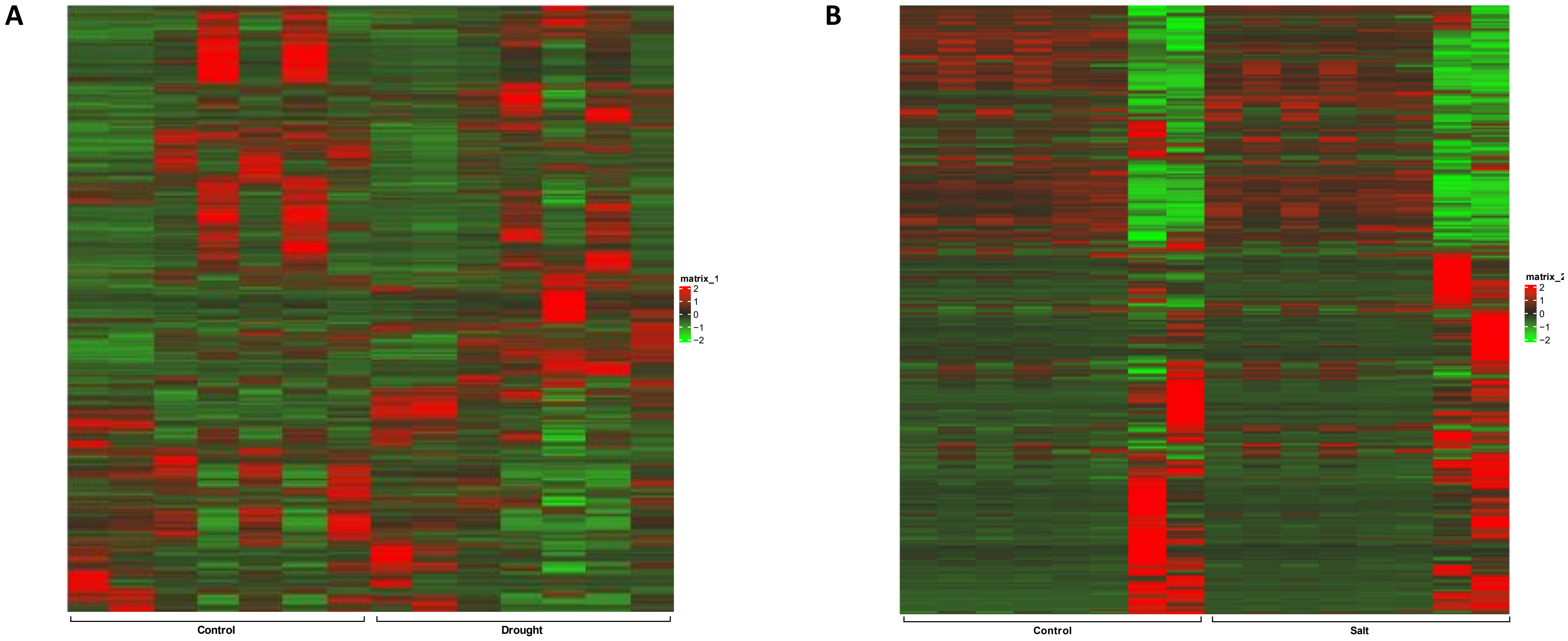

Supplementary Figure S1**.** Cluster analysis of all DEGs in different tissues under drought **(A)** and salt **(B)** treatments.

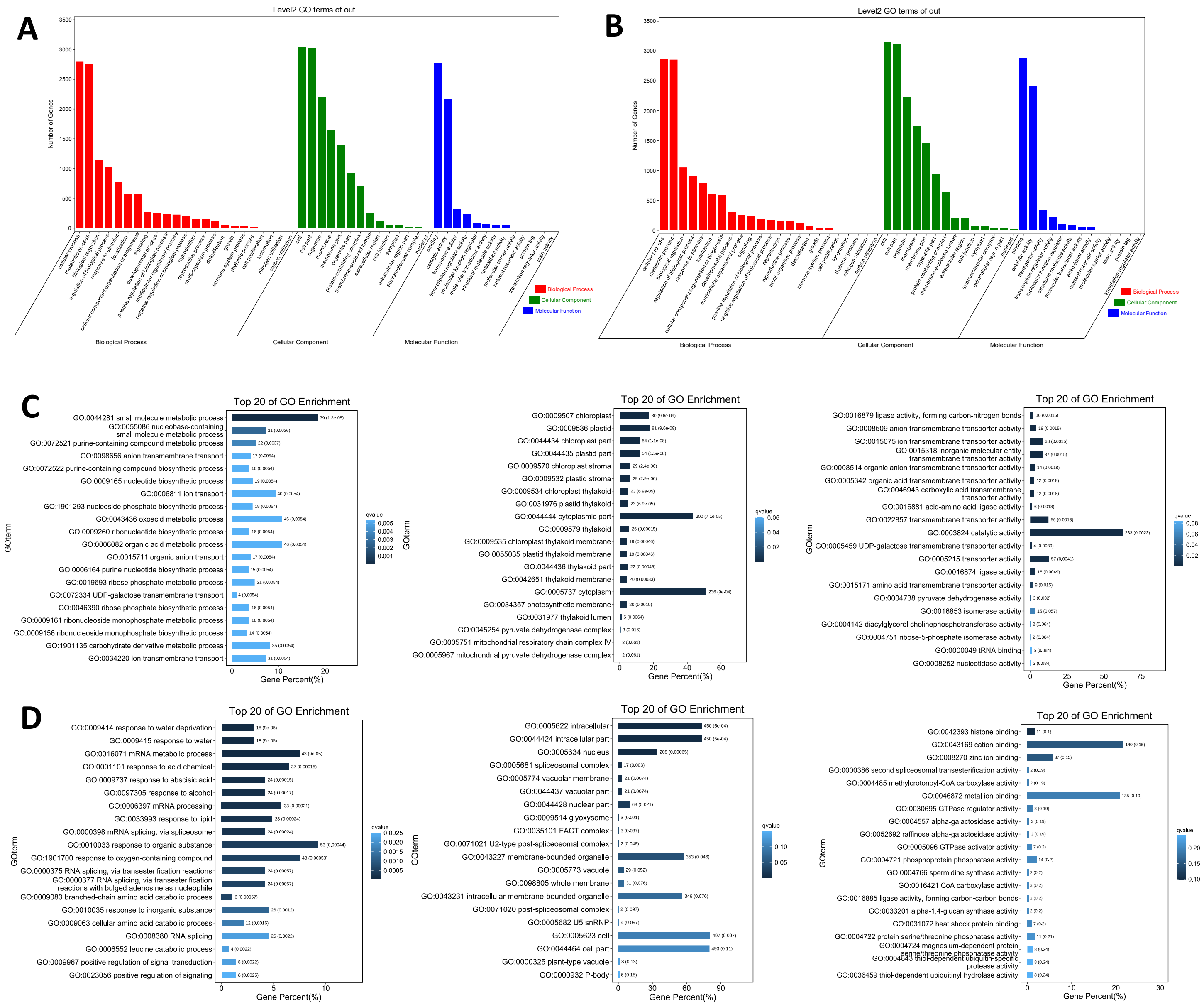

Supplementary Figure S2. **(A)** Gene ontology (GO) classification of drought-related genes. **(B)** GO classification of salt-related genes. **(C)** Significantly GO enrichment of up-regulated drought and salt co-response genes in biological process (BP), cellular part (CC) and molecular function (MF) terms. **(D)** Significantly GO enrichment of down-regulated drought and salt co-response genes in BP, CC, MF terms.

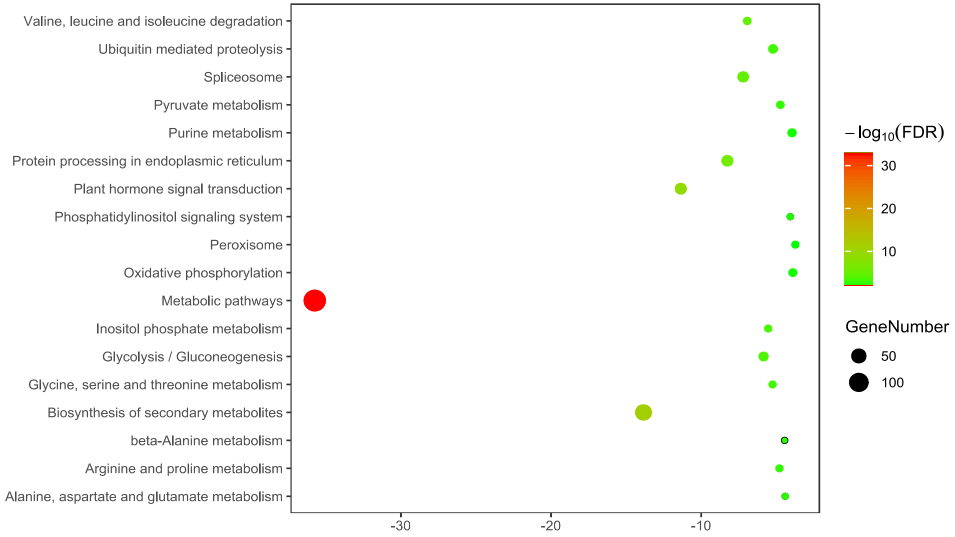

Supplementary Figure S3. KEGG analysis of drought and salt co-response genes.

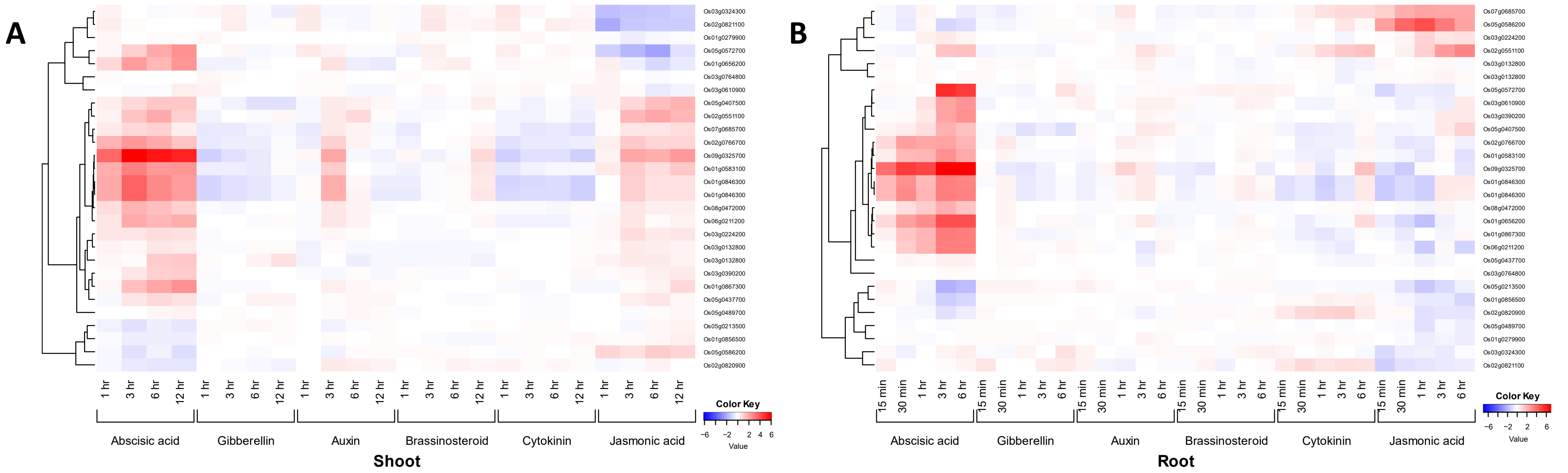

Supplementary Figure S4. The heatmap of 24 candidate genes expression under hormone treatments between **(A)** shoot and **(B)** root.

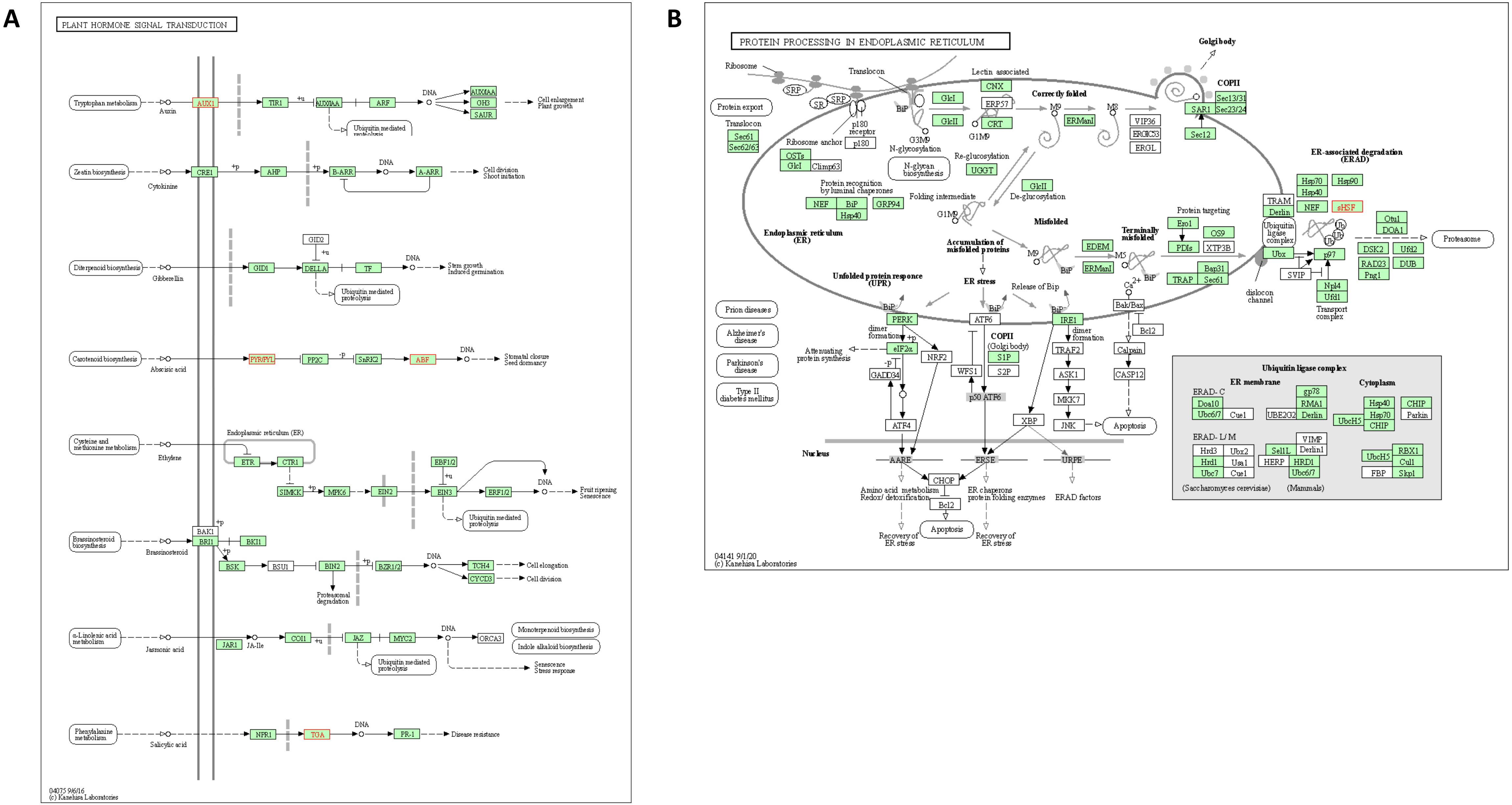

Supplementary Figure S5. KEGG pathway analysis of **(A)** osa04075 (plant hormone signal transduction) and **(B)** osa04141 (protein processing in endoplasmic reticulum).

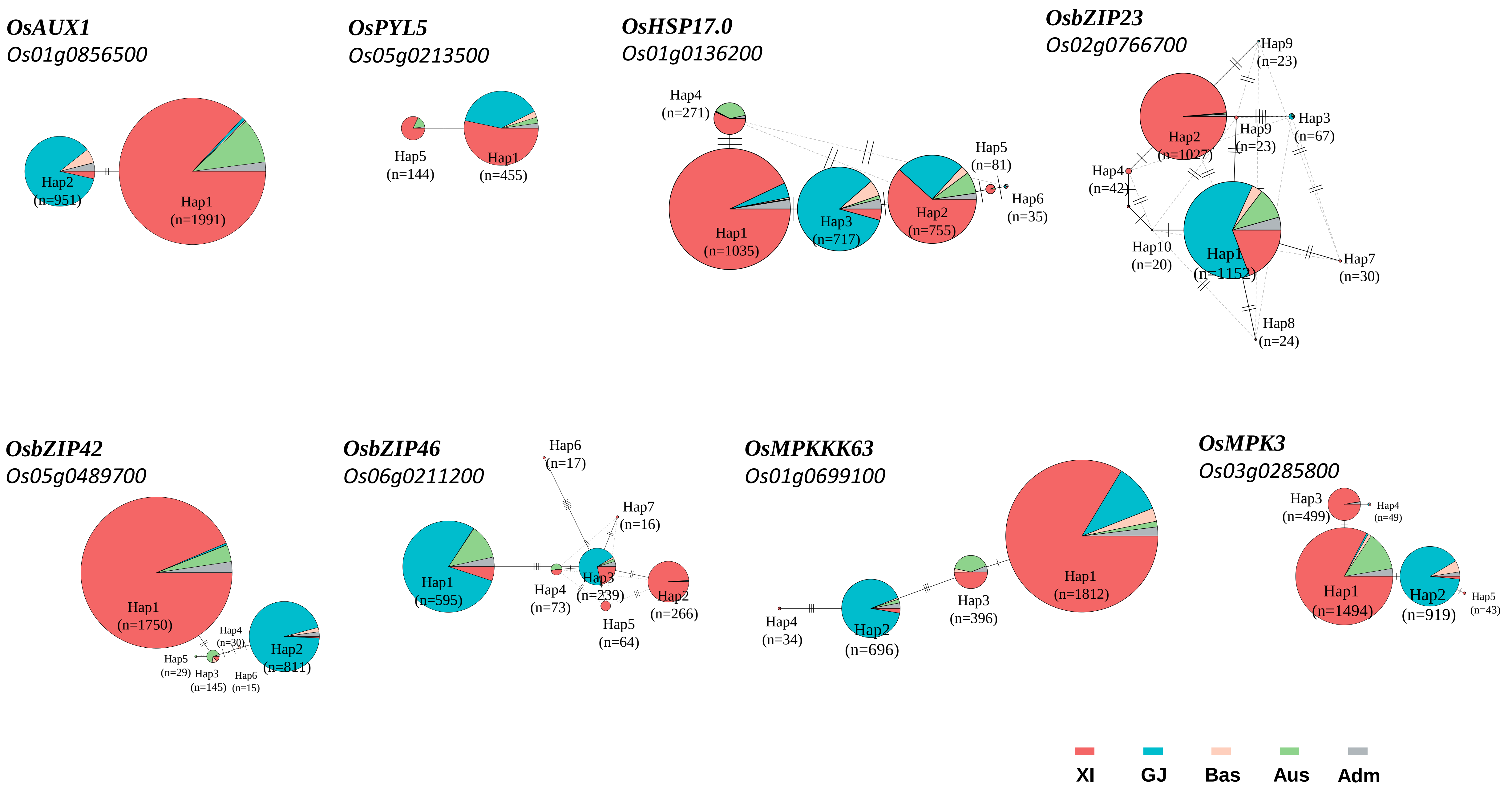

Supplementary Figure S6. Haplotype networks of seven DT and ST co-response genes in 3KRG.
